# Effects of Fasudil on Patients with Pulmonary Hypertension Associated with Left Ventricular Heart Failure with Preserved Ejection Fraction: A Prospective Intervention Study

**DOI:** 10.1155/2018/3148259

**Published:** 2018-03-26

**Authors:** Xiang Zhang, Xueming Zhang, Saihua Wang, Jun Luo, Zhihong Zhao, Changzhu Zheng, Jieyan Shen

**Affiliations:** ^1^Shanghai Zhoupu Hospital, Shanghai, China; ^2^Renji Hospital, Shanghai Jiaotong University School of Medicine, Shanghai, China

## Abstract

**Background:**

Pulmonary hypertension due to left ventricular heart failure with preserved ejection fraction (PH-HFpEF) is an increasingly medical problem. The aim of the study was to evaluate the clinical efficacy of fasudil on PH-HFpEF elderly patients and to figure out the subtype of PH-HFpEF which may be the therapeutic object of fasudil.

**Method:**

58 PH-HFpEF elderly patients were enrolled. Patients were diagnosed with passive pulmonary hypertension (PPH) or reactive pulmonary hypertension (RPH) by right heart catheterization and all receiving Rho kinase inhibitor fasudil for 2 weeks. The endpoint includes changes in SpO2, NT-pro BNP, cardiac functional classification, and echocardiography measurements after 2 weeks treatment.

**Results:**

The course of disease in the RPH group was longer than the PPH group (*p* < 0.05). Cardiac output was found to be worse in the RPH group than the PPH group (*p* < 0.01). Besides, the RPH group demonstrated a greater transpulmonary pressure gradient (TPG) and pulmonary vascular resistance (PVR) than the PPH group (*p* < 0.01 for both) as well as pulmonary arterial systolic pressure (PASP) and mean pulmonary arterial pressure (mPAP) (*p* < 0.01 for both), which fits the feature of RPH. After treatment of fasudil, in RPH group, PASP significantly decreased (*p* < 0.01) with decreased E/E′ and increased E/A (*p* < 0.05 for both), indicating that pulmonary haemodynamics and cardiac diastolic function were ameliorated, but the measurements in the PPH group had no significant changes. NT-pro BNP and 6 MWD of both groups were improved (*p* < 0.05). The total effective rate of the RPH group was 74.29%, which was higher than 47.83% of the PPH group (*p* < 0.05).

**Conclusion:**

The Rho kinase inhibitor fasudil can improve pulmonary and left ventricular haemodynamics in patients with PH-HFpEF. The total effective rate was higher in the RPH group. Fasudil may be a promising targeted drug for the RPH in PH-HFpEF patients. This trial is registered with ChiCTR-INR-16009511.

## 1. Introduction

Despite the increasing number of patients with heart failure with preserved ejection fraction (HFpEF), currently there is no proven therapy for HFpEF [1]. The long-term and sustained backward hemodynamic transmission increases the right ventricle afterload and the pulmonary artery pressure [2]. Pulmonary hypertension (PH) is recognized as one of the characteristics of HFpEF and is indeed prevalent in HFpEF patients. Thus, PH is used as a predictor of morbidity and mortality in HFpEF patients [3]. However, the optimal treatment of PH in conjunction with HFpEF is currently unknown [4].

Based on transpulmonary pressure gradient (TPG = mPAP − PAWP), pulmonary hypertension due to left heart disease (PH-LHD) could be classified into two groups: passive PH (PPH; TPG < 12 mmHg) and reactive PH (RPH, also known as the out of proportion PH; TPG ≥ 12 mmHg). The 2015 European Society of Cardiology (ESC) guidelines for the diagnosis and treatment of PH further separated PH-LHD into isolated postcapillary PH and mixed pre- and postcapillary PH. This classification was based on whether the diastolic pressure gradient (DPG = DPAP − PAWP) is lower or higher than 7 [5, 6], which is similar to the RPH and PPH classification of PH-LHD. Earlier studies had reported the roles of TPG and pulmonary vascular resistance (PVR) in predicting outcomes in heart failure. In a study of 463 patients with LV ejection fraction <40%, the mortality rate was significantly higher in patients with pulmonary vascular resistance (PVR) ≥3 WU [7], suggesting that RPH is more severe than PPH and RPH may be involve pulmonary vasculature remodeling.

Fasudil is a Rho-kinase inhibitor that blocks the activity of Rho kinase by competing the ATP binding site of the Rho-kinase catalytic domain with ATP and thus plays an important role in relaxing pulmonary vasculature. A variety of clinical studies suggested that the Rho-kinase pathway is involved in many cellular functions including proliferation, migration, and contraction of the vascular smooth muscle cell [8–10], and fasudil is considered to be a novel drug for the treatment of PH, which has been approved in Japan and China, but currently not in the US.

To date, few clinical trials of Rho kinase inhibitors have been reported in PH associated with left ventricular HFpEF. The goal of this study is to investigate the effects of fasudil on PH-HFpEF and determine the response differences to treatment between RPH and PPH.

## 2. Methods

### 2.1. Screening with Echocardiography

The study population was prospectively recruited from patients with heart failure (HF) symptoms from August 2014 to February 2017 in Zhoupu Hospital and Shanghai Renji Hospital. According to the 2016 ESC guidelines for heart failure, all symptomatic HF patients who underwent echocardiography with left ventricular ejection fraction (LVEF) ≥50% [11] were diagnosed with HFpEF. These HFpEF patients with pulmonary artery systolic pressure (PASP) >40 mmHg determined by echocardiography were suspected to be pulmonary hypertension [12].

As proposed by the ASE [13], linear internal measurements of the left ventricle and its walls are performed in the parasternal long-axis view with a two-dimensional (2D) echocardiography-guided M-mode approach, including left atrial systolic diameter (LAD), left ventricular end-diastolic diameter (LVEDD), left ventricular end-systolic diameter (LVESD), interventricular septal thickness (IVST), and left ventricular posterior wall thickness (LVPW). Fractional shortening (FS) was derived from linear measurements obtained from 2D images, and LVEF was calculated by the modified Simpson method.

Mitral valve peak E-wave velocity (*E*) and peak A-wave velocity (*A*) were obtained in the apical four-chamber view with color flow imaging for optimal alignment of pulsed-wave (PW) Doppler with blood flow. And mitral annular lateral E velocity (*E*′) was obtained in the apical four-chamber view with proper alignment to acquire septal and lateral mitral annular velocity using tissue Doppler [14]. Left ventricular diastolic dysfunction is reflected by *E*′, *E*/*A*, and *E*/*E*′.

According to ASE guidelines for the assessment of right heart [15], in the absence of a gradient across the pulmonic valve or right ventricular outflow tract, PASP is equal to right ventricular systolic pressure (RVSP). RVSP was estimated by adding transtricuspid pressure gradient to the right atrial pressure. Transtricuspid pressure gradient can be determined from the peak velocity of the tricuspid valve regurgitant jet, and right atrial (RA) pressure was estimated from IVC diameter and its collapse during inspiration under the long-axis subcostal view.

### 2.2. Confirmation with RHC and Patient Enrollment

All HFpEF patients suspected with PH by echocardiography had received optimal treatment for HFpEF and other primary diseases for 2–4 weeks, including adequate drug therapies (i.e., diuretics, nitrates, ACEI/ARB, and beta blockers) and/or interventional therapies (i.e., coronary artery stenting or pacemaker implantation). Following the above initial treatment of primary cardiac diseases, right heart catheterization (RHC) was performed, and the patients with mPAP ≥25 mmHg and PAWP >15 mmHg were confirmed as PH-HFpEF [6].

Right atrial pressure, right ventricular pressure, pulmonary artery pressure including PASP, mean pulmonary artery pressure (mPAP), diastolic pulmonary arterial pressure (DPAP), and pulmonary artery wedge pressure (PAWP) were measured by RHC using a 6-lumen Edward catheter. We replaced PAWP by LVEDP when there was PAWP measurement error caused by incomplete balloon incarceration. Cardiac output (CO) was measured in triplicate by the thermodilution technique. TPG was calculated as mPAP minus PAWP, and PVR was calculated using the following formula: (mPAP − PAWP)/CO. Among these PH-HFpEF patients, those TPG ≤12 mmHg were considered as PPH, whereas TPG >12 mmHg were classified as RPH.

The PH-HFpEF patients aged 60–80 years with HFpEF secondary to coronary disease, hypertension, degenerative valvular heart disease (except for patients with severe mitral or aortic valvular organic disease), cardiomyopathy, and New York Heart Association (NYHA) functional class II–IV were enrolled.

The exclusion criteria include the following:

1. Severe liver or kidney diseases, malignant tumor, and cerebrovascular disease
2. Restrictive cardiomyopathy, hypertrophic obstructive cardiomyopathy, constrictive pericarditis, moderate-to-severe mitral stenosis, or aortic stenosis
3. PH caused by congenital heart disease, rheumatic heart disease, autoimmune disease, chronic obstructive pulmonary disease, chronic thromboembolism, pulmonary vascular abnormalities, and idiopathic PAH (IPAH)
4. Patients who are treated with calcium blockers, prostacyclin, or endothelin receptor antagonists
5. Patients with incomplete data

A total of 58 PH-HFpEF patients were enrolled, of which 35 were diagnosed with RPH while 23 patients were confirmed as PPH.

### 2.3. Treatment and Efficacy Evaluation

These enrolled patients were treated with fasudil (fasudil injection solution from the Tianjin Hongri Pharmaceutical Ltd by Share Ltd), 30 mg intravenous drip twice a day for 2 weeks. During the treatment, the heart rate, blood pressure, liver function, renal function, and electrolytes were closely monitored in case adverse events or background therapy remained unchanged.

Data collection was undertaken and compared pre- and posttreatment results in all patients, including Doppler echocardiography, measurement of 6MWD, NYHA heart failure functional classification, and laboratory tests results, such as NT-pro-BNP and SvO_2_.

We evaluated the cardiac function of patients based on NYHA classification and 6-minute walking distance (6MWD). The efficacy of fasudil was determined according to the changes of NYHA classification: (1) markedly effective: heart failure is basically controlled or cardiac functional classification increased by 2 and above; (2) effective: cardiac functional classification increases by 1, but less than 2; (3) ineffective: cardiac function is better than before, but its classification did not change; and (4) deterioration: cardiac functional classification decreases by 1 or more.

### 2.4. Statistical Analyses

Continuous variables are expressed as mean ± SEM unless otherwise stated. The differences of baseline and baseline changes between RPH and PPH were compared using Student's *t*-test for normally distributed data, and the differences of examination data between pre- and posttreatment were evaluated using the paired *t*-test. The Wilcoxon test was used for continuous nonnormally distributed variables. Enumeration data were presented in terms of the rate and test by χ^2^ or exact probability method, and the rank sum test was used for ranked data.

Two-sided *p* value < 0.05 was considered to indicate statistically significant. Statistical analyses were performed using SAS and SPSS version 17.0.

## 3. Results

### 3.1. Baseline Characteristics and Pulmonary Hemodynamics by RHC

The patients' demographic and etiological data and pulmonary hemodynamic measurements, as well as the medication used for basic diseases and heart failure treatment, are summarized in Table 1. Baseline characteristics of the two groups were comparable except for the course of disease and pulmonary hemodynamics.

The disease course in the RPH group was longer than that in the PPH group (3.69 ± 2.64 years versus 3.09 ± 2.22 years, *p* < 0.05), and the TPG and PVR in the RPH group were higher than those in the PPH group (TPG: 22.11 ± 2.25 mmHg versus 10.08 ± 1.88 mmHg, *p* < 0.01; PVR: 5.24 ± 2.55 WU versus 2.15 ± 0.88 WU, *p* < 0.01), which are the characteristics of patients with RPH. Meanwhile, compared to the PPH group, PASP and mPAP in RPH patients were also higher (PASP: 62.9 ± 17.47 mmHg versus 47.17 ± 8.47 mmHg, *p* < 0.01; mPAP: 45.26 ± 14.96 mmHg versus 33.82 ± 7.26 mmHg, *p* < 0.01) while CO was lower (4.19 ± 1.26 versus 4.84 ± 1.56, *p* < 0.01), suggesting that pulmonary hypertension in RPH patients is more severe than that in PPH and leads to CO decrease.

### 3.2. Echocardiography

As shown in Table 2, there was no significant difference in echocardiography baselines between RPH and PPH groups except for PASP (64.37 ± 13.82 versus 49.87 ± 8.50 mmHg, *p* < 0.05).

After 2 weeks of therapy with fasudil, the left ventricular diastolic function in RPH group, as indicated by decreased *E*/*E*′ and increased *E*/*A*, was improved (*E*/*E*′ 16.89 ± 1.11 versus 13.19 ± 1.79, *p* < 0.05; *E*/*A* 0.78 ± 0.19 versus 0.98 ± 0.12, *p* < 0.05), and PASP in the RPH group significantly decreased from 64.37 ± 13.82 mmHg to 63.51 ± 13.79 mmHg (*p* < 0.05). However, there was no change in *E*/*E*′, *E*/*A*, and PASP in the PPH group.

### 3.3. Cardiac Function Evaluation and Laboratory Tests

At baseline, there were no statistical differences between the study groups in SvO_2_, NT-pro-BNP, 6MWD, and NYHA classification.

**Figure 1:**
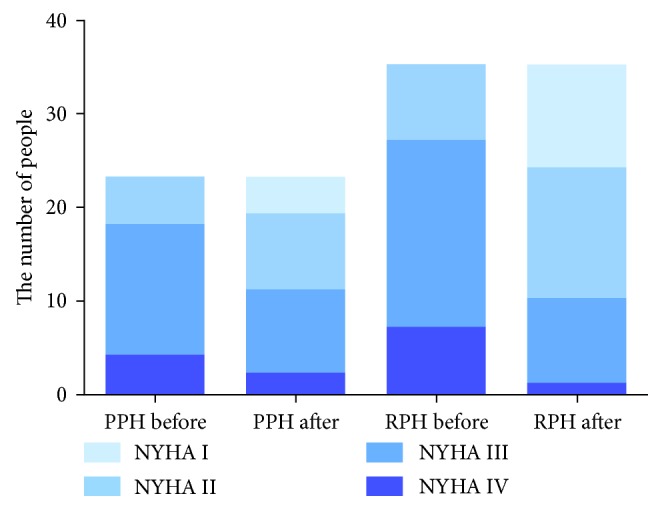
Fasudil treatment-induced changes of patient's NYHA classification in the RPH and PPH groups.

In both groups, NT-pro-BNP and 6MWD were improved after treatment as summarized in Table 3(*p* < 0.05). In terms of NYHA classification, the RPH group had a better outcome in comparison to the PPH group: 8 cases of the RPH group were markedly effective, effective in 18 cases, and invalid or deteriorated in 9 cases. The total effective rate in RPH group was 74.29%, whereas the total effective rate was 47.83% in the PPH group including markedly effective in 2 cases, effective in 9 case, and invalid or deteriorated in 12 cases (Table 4, Figure 1). Fasudil was more effective for RPH patients, indicated by a significant difference in the total effective rate between the two groups (*p* < 0.05).

### 3.4. Safety Analysis

During the 2-week treatment, no adverse event was observed in either group; patient's liver function, renal function, electrolyte, blood, urine, and stool routine tests were all normal. Two patients in the PPH group and 3 patients in the RPH group reported some mild side effects including slightly decreased heart rate and blood pressure, dizziness, and fatigue, which were all reported tolerable to the patients and thus fasudil treatment was not discontinued.

## 4. Discussion

Heart failure with preserved ejection fraction originates from left ventricular diastolic dysfunction secondary to impaired relaxation and stiffened myocardium. The increased load of left ventricle exposes the lung vasculature to pressure-induced challenges. In the long term, the sustained pulmonary hemodynamic change not only leads to pulmonary hypertension but also increases the pulsatile loading on the right ventricle [2], which determines the patient's outcome.

The management of pulmonary hypertension due to left heart disease (PH-LHD) focuses on treating the primary left heart disease and improves left ventricular function, thereby alleviating PH. However, even with optimal treatment, the clinical symptoms and mortality of this disease cannot be improved. Aronson et al. [16] performed a survival analysis of 242 patients with acute decompensated heart failure and observed that the mortality of RPH patients was significantly higher than those of PPH patients or patients without PH. Meanwhile, a population-based report by Lam et al. [3] revealed that, in hypertensive patients with HFpEF, the increased pulmonary artery pressure may not be attributable to the passive venous PH and there may be an additional precapillary pulmonary artery hypertension mediated by functional or organic arteriolar disorders, which may need a novel therapeutic strategy for PH-HFpEF.

Recently, whether PH caused by pulmonary vascular remodeling in late PH-LHD can be treated with targeted drugs has drawn much attention. FIRST [17] and ENABLE [18] studies proved that pulmonary vascular targeting therapy is not suitable for PH due to left heart failure. However, these studies had selected the patients with severe left ventricular systolic dysfunction without evidence of PVR elevating, so these results are not enough powerful to prove that pulmonary vascular targeting therapy is not effective for RPH-HFpEF on the basis of optimal treatment for HFpEF. A previous study of sildenafil [19] effects on PH-HFpEF proved that sildenafil improves pulmonary pressure, vasomotility, and RV function, as well as LV relaxation and distensibility. However, this study did not perform right heart catheterization and subgroup PVR analysis. Recently, there were two randomized placebo-controlled trials of sildenafil's effects in patients with HFpEF and predominantly isolated postcapillary PH reported by Hoendermis et al. [20] and Liu et al. [21], respectively. Both trials showed similar results suggesting that sildenafil is ineffective in improving pulmonary and systemic hemodynamics. Currently, there is little evidence on target therapy for RPH-HFpEF.

The results of our current prospective controlled trial show that fasudil can reduce pulmonary artery pressure and improve cardiac function more significantly in RPH-HFpEF than in PPH-HFpEF patients. We found that RPH patients had a longer course of heart failure, higher levels of PASP, mPAP, TPG, and PVR than those of PPH patients. These findings may be explained by the hypothesis that RPH and PPH have different pathophysiology, and PPH and RPH may be two different stages of PH-HFpEF. PPH is the early stage due to passive transduction of the increased pulmonary venous pressure, whereas RPH is the advanced stage characterized by vascular constriction and proliferation caused by chronic progressive increase of pulmonary artery wedged pressure and pulmonary artery pressure, which leads to the elevation of TPG and PVR, as well as pulmonary vasculopathy. In contrast, there was no significant difference between RPH and PPH in NT-pro-BNP, 6MWD, NYHA, and other cardiac functional indexes suggesting that the cardiac functional impairment of RPH might not be proportional to the increase of pulmonary artery pressure.

Fasudil is a potent selective inhibitor of Rho kinase. Several trials studying the acute- or long-term effects of fasudil on PAH have demonstrated that fasudil improves pulmonary hemodynamics [22–24], which is consistent with our finding that fasudil reduced pulmonary artery systolic pressure in the RPH group. In contrast, pulmonary hemodynamic in the PPH group had no significant change. In terms of cardiac function, there is a significant posttreatment improvement in both groups with higher overall effectiveness in the RPH group.

In animal studies, fasudil was reported to decrease the mean pulmonary arterial pressure, right ventricular hypertrophy, and pulmonary arteriolar medial thickness and augment pulmonary expression of endothelial nitric oxide synthase (eNOS) in rats with PH secondary to left ventricular dysfunction [25]. Furthermore, fasudil was also reported to attenuate pulmonary arteriole endothelial cell injury and the proliferation of smooth muscle cells and collagen fibers [26], as well as lessened the expression of thioredoxin-1 (Trx-1) and hypoxia inducible factor-1α (HIF-1*α*) [27] in monocrotaline or chronic hypoxia-induced rat PH models. Our findings demonstrated the beneficial effects of fasudil in RPH patients that could be explained by alleviation of pulmonary vasculopathy of RPH-HFpEF and reduction of PVR and PASP.

With regard to the left heart, Guo et al. [28] reported that fasudil improves short-term echocardiographic parameters of left ventricular diastolic function in patients with type-2 diabetes with preserved left ventricular ejection fraction, which is consistent with our results that fasudil therapy leads to improvement in diastolic function as reflected by increase in *E*/*A* ratio and decrease in *E*/*E*′. Several lines of evidence have revealed that Rho/ROCK inhibitors not only increase eNOS expression both in vivo and in vitro but also regulate the activity of myosin phosphatase activity by inducing the phosphorylation of its myosin-binding subunit [9, 29], which may affect left ventricular (LV) relaxation and LV filling pressure. Moreover, the changes in LV diastolic function may result from the interaction between the LV and RV. Due to the PAH, the distension of patient's RV and atrium may impair LV relaxation and compliance properties. Fasudil may help relieve the compression of the LV by reducing right heart pressure and volumes. Further trials are needed to identify the precise effect of fasudil on LV diastolic function.

In terms of study limitations, the main limitations of the current study include the relatively small number of trial size, the lack of RHC reexamination, and the data of RV function improvement, which limit the precision for estimation of the magnitude of effects. In addition, there is no placebo control group in this study which restricts the accuracy of the efficacy estimation of fasudil. Lastly, the long-term effect of fasudil on RPH or PPH-HFpEF is still unknown.

In summary, Newman et al. had proposed that redefining pulmonary hypertension through pulmonary vascular disease phenomics and resolving the heterogeneity of the PH syndrome will allow for more targeted therapeutics [30]. PH-LHD is also a complex clinical syndrome with complex pathophysiology, and precision medicine indication may have a good therapeutic effect on PH-LHD. Our study provides evidence that fasudil has a better therapeutic effect on RPH-HFpEF than PPH suggesting that fasudil could be an effective precise treatment of RPH-HFpEF. A multicenter, randomized, placebo-controlled trial with optimizing inclusion criteria and dose selection is needed to further investigate the efficacy and safety of fasudil as a potential targeted drug therapy in PH-LHD.

## Figures and Tables

**Table 1:** Baseline characteristics and pulmonary hemodynamics by RHC.

Variable	PPH group (*n* = 23)	RPH group (*n* = 35)	*p*
*Demographics*			
Male, *n* (%)	16 (69.57)	23 (65.71)	0.783
Female, *n* (%)	7 (30.43)	12 (34.29)	0.684
Age (years)	69.78 ± 10.22	70.60 ± 11.14	0.891

*Medical history*			
Coronary artery disease (%)	9 (39.13)	13 (37.14)	0.965
Hypertensive heart disease (%)	2 (8.70)	3 (8.57)	0.974
Coronary heart disease with hypertension (%)	7 (30.43)	10 (28.57)	0.862
Dilated cardiomyopathy (%)	2 (8.69)	3 (8.57)	0.975
Degenerative valvular heart disease (%)	3 (13.04)	6 (17.14)	0.763
Course of disease (years)	3.09 ± 2.22	3.69 ± 2.64^∗^	0.012

*Medication, n (%)*			
Diuretics	22 (95.65)	33 (94.29)	0.912
Hydrochlorothiazide 25 mg qd	15	24	
Torasemide 20 mg qd	7	9	
Antisterone	22	31	
Nitrates	17 (73.91)	26 (74.29)	0.893
ACEI/ARB	19 (82.61)	29 (82.86)	0.981
*β*-Blockers	20 (86.96)	28 (80.00)	0.261

*Pulmonary hemodynamics*			
PASP (mmHg)	47.17 ± 8.47	62.9 ± 17.47	0.003
mPAP (mmHg)	33.82 ± 7.26	45.26 ± 14.96	0.007
PAWP (mmHg)	23.26 ± 5.26	23.14 ± 5.26	0.947
TPG (mmHg)	10.08 ± 1.88	22.11 ± 2.25	0.000
PVR (WU)	2.15 ± 0.88	5.24 ± 2.55	0.003
CO (L/min)	4.84 ± 1.56	4.19 ± 1.26	0.000

*Cardiac function (n/n)*			
NYHA I-II/III-IV	5/18	8/27	0.9426

Data are presented as number and rate or mean ± standard deviation; Student's *t*-test was used for continuous normally distributed variables; the Wilcoxon test was used for continuous nonnormally distributed variables; ranked data were tested by rank sum test; PASP, pulmonary artery systolic pressure; mPAP, mean pulmonary artery pressure; PAWP, pulmonary artery wedge pressure; TPG, transpulmonary pressure gradient; PVR, pulmonary vascular resistance; CO, cardiac output; NYHA, New York Heart Association; the change from baseline, ^∗^*p* < 0.05.

**Table 2:** Cardiac effect of fasudil treatment determined by echocardiography.

Variable	PPH (*n* = 23)			RPH (*n* = 35)		
	Pretreatment	Posttreatment	*p*	Pretreatment	Posttreatment	*p*
LAD (mm)	46.50 ± 4.75	45.50 ± 4.51	0.987	47.00 ± 6.25	46.63 ± 5.50	0.379
LVEDD (mm)	45.10 ± 6.14	46.07 ± 8.12	0.697	47.14 ± 8.40	47.63 ± 7.73	0.497
LVESD (mm)	27.91 ± 3.16	28.21 ± 2.88	0.378	29.02 ± 3.64	29.91 ± 4.06	0.788
IVST	11.01 ± 1.56	10.98 ± 1.96	0.916	11.85 ± 2.03	11.35 ± 1.96	0.895
LVPW	10.82 ± 1.16	10.01 ± 0.96	0.816	11.01 ± 1.76	10.87 ± 1.86	0.941
*E* (cm/s)	49.97 ± 6.85	52.59 ± 8.37	0.062	50.35 ± 8.22	53.43 ± 9.22	0.059
*A* (cm/s)	62.03 ± 10.31	63.36 ± 9.37	0.096	64.5 ± 11.80	54.52 ± 12.65^∗^	0.016
*E*′ (cm/s)	2.89 ± 1.03	3.03 ± 1.11	0.074	2.98 ± 1.16	4.05 ± 1.02	0.071
*E*/*A*	0.80 ± 0.11	0.83 ± 0.14	0.069	0.78 ± 0.19	0.98 ± 0.12^∗^	0.011
*E*/*E*′	17.29 ± 2.21	17.36 ± 1.89	0.098	16.89 ± 1.11	13.19 ± 1.79^∗^	0.011
EF (%)	61.64 ± 10.31	64.83 ± 12.37	0.069	62.43 ± 11.80	64.24 ± 9.65	0.485
PASP (mmHg)	49.87 ± 8.50	49.48 ± 8.64	0.444	64.37 ± 13.82^†^	63.51 ± 13.79^∗^	0.017

Data are presented as mean ± standard deviation; differences in change from baseline between groups were evaluated using Student's *t*-test; LAD, left atrial systolic diameter; LVEDD, left ventricular end-diastolic diameter; LVESD, left ventricular end-systolic diameter; IVST, interventricular septal thickness; LVPW, left ventricular posterior wall thickness; E, early diastolic transmitral flow velocity; A, late diastolic transmitral flow velocity; *E*′, early diastolic mitral annular velocity; ^†^the comparison of PASP between groups before treatment, *p* < 0.01; the change from baseline, ^∗^*p* < 0.05.

**Table 3:** Effect of fasudil on SpO_2_, NT-pro-BNP, and 6MWD.

Variable	PPH (*n* = 23)			RPH (*n* = 35)		
	Pretreatment	Posttreatment	*p*	Pretreatment	Posttreatment	*p*
SpO_2_ (%)	92.89 ± 3.97	93.15 ± 5.13	0.071	91.30 ± 4.68	93.07 ± 4.80	0.052
NT-pro-BNP (ng/ml) (min, max)	3664 (789, 21150)	1149 (750, 4651)^∗^	0.031	3980 (1312, 24000)	1287 (155, 8210)^∗∗^	0.004
6MWD (m)	361.75 ± 50.24	401.34 ± 59.56^∗^	0.042	345.60 ± 44.55	400.7 ± 59.41^∗∗^	0.001

Normally distributed data are presented as mean ± standard deviation; nonnormally distributed data are presented as minimum and maximum; normally distributed variables were evaluated using Student's *t*-test; nonnormally distributed data were tested by the Wilcoxon test;; 6MWD, 6-minute walking distance; the change from baseline,^∗^*p* < 0.05  and  ^∗∗^*p* < 0.01.

**Table 4:** The number of cases of NYHA classification change.

Efficacy	PPH (*n* = 23)	RPH (*n* = 35)	*p*
Markedly effective (%)	2 (8.70)	8 (22.86)	
Effective (%)	9 (39.13)	18 (51.43)	
Ineffective or deterioration (%)	12 (52.17)	9 (25.17)	
Total effective (%)	11 (47.83)	26 (74.29)	0.040

Data are presented as patient number and %; enumeration data are tested by *χ*^2^ test or exact probability method.
